# Increased risk of hepatic complications in kidney transplantation with chronic virus hepatitis infection: A nationwide population-based cohort study

**DOI:** 10.1038/srep21312

**Published:** 2016-02-19

**Authors:** Tung-Min Yu, Che-Chen Lin, Kuo-Hsiung Shu, Ya-Wen Chuang, Shih-Ting Huang, Cheng-Hsu Chen, Ming-Ju Wu, Mu-Chi Chung, Chao-Hsiang Chang, Chi-Yuan Li, Chi-Jung Chung

**Affiliations:** 1 Graduate Institute of Clinical Medical Science, College of Medicine, China Medical University, Taichung, Taiwan; 2 Division of Nephrology, Taichung Veterans General Hospital, Taichung, Taiwan; 3 Department of Medicine, College of Medicine, China Medical University, Taichung, Taiwan; 4 Management Office for Health Data, China Medical University Hospital, Taichung, Taiwan; 5 Department of Urology, China Medical University and Hospital, Taichung, Taiwan; 6 Department of Anesthesiology, China Medical University Hospital, Taichung, Taiwan; 7 Department of Health Risk Management, College of Public Health, China Medical University, Taichung, Taiwan; 8 Department of Medical Research, China Medical University Hospital, Taichung, Taiwan

## Abstract

Data regarding the risk of various liver diseases among different hepatitis viruses in kidney transplantation have not yet been identified.We selected individuals with kidney transplantation (ICD-9-CM V420 or 996.81) from 2000–2009 from the catastrophic illness registry of National Health Insurance Research Database (NHIRD)as the study cohort. The two end-points in the study included overall death, and post-transplant occurrence of hepatic disease. After adjustment for other risk factors, the risk of mortality was increased in patients with HBV infection (N = 352) and with HCV infection (N = 275) compared to those with neither HBV nor HCV infection (N = 3485). In addition,renal transplant recipients with HBV alone,HCV alone, and both with HBV and HCVinfectionrespectively had an approximately 10-fold hazard ratio (HR) = 9.84, 95% confidence interval (CI): 4.61–21.0, 4-fold increased risk (HR = 4.40, 95% CI: 1.85–10.5)and 5-fold increased risk (HR = 4.63, 95% CI: 1.06–20.2)of hepatocellular carcinoma (HCC)compared to those with neither HBV nor HCV infection. Our findings showed a significant risk of de novo liver disease in recipients with hepatitis virus infection. Based on our findings, we reinforce the importance and impact of hepatitis virus in renal transplantation.

Kidney transplantation has been considered a better choice of treatment for patients with end-stage renal disease (ESRD), as compared to patients receiving maintenance dialysis. Meanwhile, a growing body of evidence is showing that hepatitis virus infection frequently coexists with ESRD patients and may adversely affect long-term outcomes with regards to kidney transplantation.

Regardless of the condition of renal transplant recipients or dialysis patients, chronic infection with hepatitis virus, mainly hepatitis B virus (HBV) and hepatitis C virus (HCV), is more prevalent in ESRD patients than in the general population. The prevalence rate of HBV infection has been estimated to be around 0–10% in dialyzed patients, and may be as high as approximately 20% in some developing countries^1^. For example, HBV was reported to be as high as 14% in ESRD populations in some endemic Asia-Pacific countries^2^,^3^. It is true as well that HCV infection remains highly prevalent in both developed and less-developed countries among ESRD patients^4^,^5^. The prevalence of HCV infection in dialyzed patients ranged between 10–65%, and that inkidney transplant recipients this has been estimated to be between 6–46%^1^,^3^.

Post-transplantation liver disease is suggested to be associated with adverse outcomes in kidney transplantation which was considered as the fourth most important cause of mortality in kidney recipients^1^,^6^,^7^. Hepatitis virus infection is thought to play an important role in post-transplant liver disease. Both HBV and HCV infection have been suggested as being associated with progressive liver diseases after transplantation, including liver cirrhosis, hepatocellular carcinoma (HCC) and hepatic failure. In a study of kidney transplantation with HBV infection, 85% of individuals had liver progression, with chronic activehepatitis in up to 42% and cirrhosis in 28%^3^,^8^. This is similar as well in cases with HCV infection which a lower viremia and a lower rate of cirrhosis (10% versus 25–40%) were found in dialysis patients with HCV as compared to renal transplant recipients^1^.

Although the influence of virus hepatitis in ESRD patients is a concern, there is still the matter of controversy in previous studies. The natural course of hepatitis virus in kidney transplantation is more complex than non-transplant patients and would inevitably be adversely affected by multiple factors, in particular the accumulative effect of immunosuppression. It is believed that immunosuppression could promote viral replication in cisternae of the endoplasmic reticulum in hepatocytes and enhance the progression of quiescent liver diseases to cirrhosis and HCC, which would eventually result in higher liver disease-related mortality in kidney recipients than in those without hepatitis virus^1^. As a whole, hepatitis virus and immunosuppression in kidney transplantation is suggested to involve in mediating post-transplant liver disease; however, data to elucidate the relationship between virus hepatitis, immunosuppression and risk of various post-transplant liver diseases is relatively limited.

Taiwan is an endemic region for the hepatitis virus and the prevalence of HBV and HCV is considerably higher, reaching approximately 10% among ESRD patients^2^. A remarkably high prevalence of chronic hepatitis virus infection is therefore found in kidney transplant patients, with HBV infection reaching approximately 20.9% and HCV, 46.3%, respectively^4^. With these findings, we are striving to determine the long-term outcomes of kidney transplant patients with hepatitis virus infection and further clarify the relationship between the hepatitis virus, immunosuppression and risk of individual subtype liver disease after transplantation in a nationwide cohort study.

## Methods

### Data Source

In 1995, the Taiwan government implemented a single-payer universal health insurance system, the Taiwan National Health Insurance (NHI) program, which covers more than 99% of the 23 million residents in Taiwan. The National Health Research Institute (NHRI) has compiled annual claims data from the NHI program, encoded personal identification information, and released the database for research purposes. Data for our cohort study were obtained from the National Health Insurance Research Database (NHIRD), which is comprised of comprehensive information on the clinical visits for each insurant, including demographic data, date of visits and medical services. In order to link each person’s data, the NHRI provided a scrambled and anonymous identification number.

The International Classification of Disease, 9th Revision, Clinical Modification (ICD-9-CM) coding system was used for disease data in the NHIRD. The disease history was collected from the catastrophic illness registry and inpatient files.

### Study population

This study used a population-based retrospective cohort study design. We selected individuals in the catastrophic illness registry who had undergone kidney transplantation (ICD-9-CM V420 or 996.81) from 2000–2009 as the study cohort. The kidney transplantation cohort was separated into 4 groups based on the type of hepatitis infection before hepatic disease occurrence: those without HBV and HCV infection as Group 1; those with HBV infection only as Group 2; those with HCV infection only as Group 3; and those both with HBV and HCV infection as Group 4. We excluded those with pre-existing hepatic diseases before kidney transplantation and those with HBV and HCV co-infection simultaneously. In Taiwan, ESRD patients annually received routine liver examinations including abdominal images such as ultrasonography or computer tomography and serum biochemistry (GOT/GPT, albumin, alpha-fetoprotein, hepatitis B and C virus markers, et ac) while on waiting list and at the time before transplant operation.

We observed two end-points: 1) death, and 2) post-transplant occurrence of hepatic diseases (ICD-9-CM 155.0, 570 and 571.5). Hepatic disease occurrence was identified as of 3 subtypes: HCC (ICD-9-CM 155.0), hepatic failure (ICD-9-CM 570) and liver cirrhosis (ICD-9-CM 571.5). The follow-up of the study population was terminated when the subject withdrew from the insurance program,event occurrence, death or on December 31, 2010.

Comorbidities were also considered as confounding factors. These included diabetes mellitus (DM, ICD-9-CM 250), hypertension (ICD-9-CM 401–405), heart failure (ICD-9-CM 428), coronary heart diseases (ICD-9-CM 410–414), and CGN (ICD-9:581, 582, 583), as found in the inpatient files.

### Statistical analysis

We presented the mean and standard deviation (SD) for continuous variables and number and proportion for categorical variables. To assess the distribution difference in the study groups, we used the chi-square test for categorical variables and analysis of variance (ANOVA) for continuous variables. Mortality and overall hepatic disease incidence in the study groups were calculated as the total number of events (death or overall hepatic disease occurrence) divided by the total number of follow-up years for each group (per 1000 person-years). The Kaplan-Meier method was used to calculate survival curves and cumulative incidence curves. The risk of mortality and developing hepatic disease was calculated using the multivariable Cox proportional hazard model and presented using hazard ratios (HRs) and 95% confidence intervals (CIs).

A two-tailed p value of <0.05 was considered statistically significant. All statistical analyses were performed with SAS statistical software (version 9.3 for Windows; SAS Institute, Inc., Cary, NC, USA). Survival curves and cumulative incidence curves were plotted with SPSS.

## Results

The 4,133 kidney recipients were divided into three groups: 3485 patients with neither HBV nor HCV infection (Group 1), 336 (8.13%) with HBV infection (Group 2), 262 (6.34%) with HCV infection (Group 3) and 50 (1.21%)both with HBV and HCV infection (Group 4) enrolled into the study (Table 1). More than half of the patients were males and had undergone kidney transplantation from 2000 to 2005.

The mean age at kidney transplantation was 46.9 years in Group 3, 44.6 years in Group 2 and 45.8 years in Group 1, which was statistically significant (p < 0.0446). More patients had diabetes mellitus in Group 3 than in the other groups, which was a significant difference (p < 0.001). Other cardiovascular risks such as hypertension, coronary artery disease and congestive heart failure were comparable among all groups and were not statistically different (Table1).

To estimate the impact of hepatitis virus infection on overall mortality among kidney recipients, multiple factors that were relevant to patient survival were calculated using the Cox-regression model. After adjustment for age, sex, DM, hypertension, CGN, heart failure and CAD, the risk of virus hepatitis in patient survival showed an adjusted HR = 2.99, 95% CI: 2.13 to 4.18 in transplant recipients with HBV infection, an adjusted HR = 2.05, 95% CI: 1.52 to 2.76 in those with HCV infection and an adjusted HR = 1.36, 95% CI: 0.61 to 3.07 in those both withHBVand HCV infection (Table 2). The risks of different subtype of hepatic diseases varied greatly among the three cohorts. Compared to Group 1, HBV patients had an approximately 10-fold increased risk of HCC (aHR = 9.84, 95% CI: 4.61 to 21.0), HCV patients showed an aHR = 4.40, 95% CI: 1.85 to 10.5 and both HBV and HCV patients showed anaHR = 4.63, 95% CI: 1.06 to 20.2. The risk of liver cirrhosis in HCV patients showed an aHR = 18.0, 95% CI: 9.78 to 33.2, and that in HBV cases showed an aHR = 5.86, 95% CI: 2.42 to 14.2. With regards to the condition of fulminant liver failure, HBV cases had an aHR = 5.63, 95% CI: 2.47 to 12.8 in hepatic failure, HCV cases had an aHR = 2.06, 95% CI: 0.71 to 6.0, and both HBV and HCV cases had an a HR = 4.6, 95% CI: 1.05 to 20.1, which did not achieve a statistical difference (Table 2).

**Figure 1:**
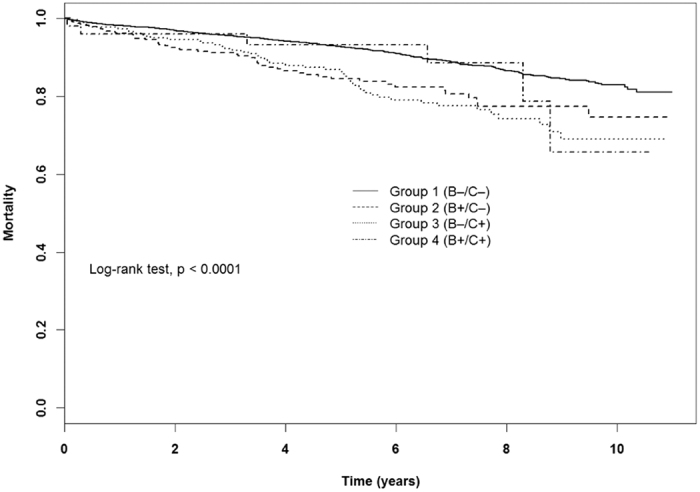
Comparison of patient survival stratified by virus status.

**Figure 2:**
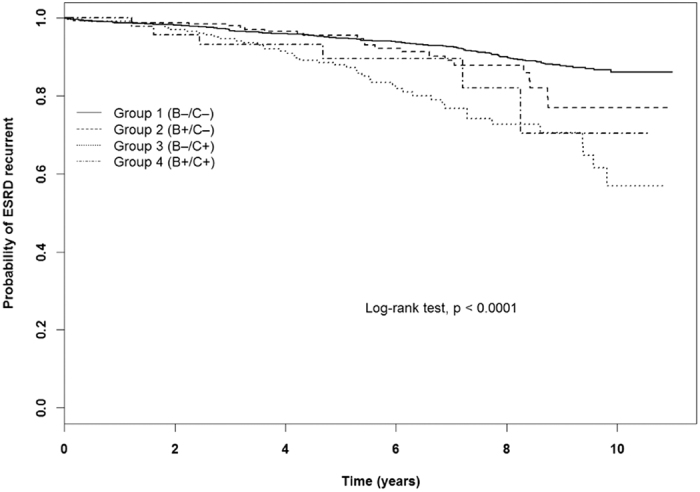
Comparison of kidney graft survival stratified by virus status.

**Figure 3:**
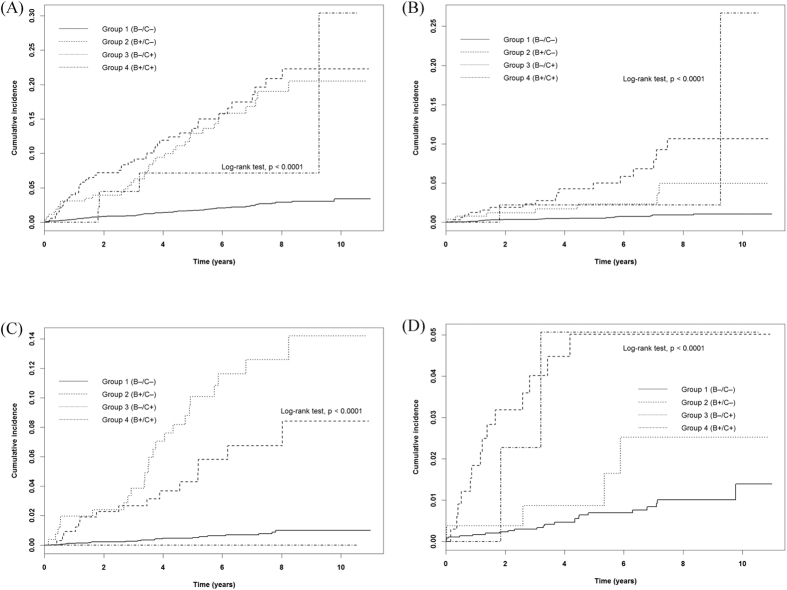
Cumulative incidence rates of subsequent hepatic diseases stratified by virus status: (**A**) overall hepatic diseases, (**B**) hepatocellular carcinoma, (**C**) liver cirrhosis, and (**D**) hepatic failure.

Other risk factors contributing to patient survival included age >65 years (aHR = 5.63, 95% CI: 3.74 to8.48); diabetes mellitus (aHR = 1.82, 95% CI: 1.47 to 2.26); congestive heart failure(aHR = 1.81, 95% CI: 1.33 to 2.46); and coronary artery diseases(aHR = 1.99, 95% CI: 1.52 to 2.61). In kidney recipients, the risk factors predicting the occurrence of de novo hepatic diseases included HBV(aHR = 6.76, 95% CI: 4.24 to 10.8), HCV(aHR = 6.57, 95% CI: 4.31 to 10.0), male(aHR = 1.83, 95% CI: 1.30 to 2.58),aged between 40and65 years (aHR = 2.69, 95% CI: 1.73 to 4.18) and aged older than 65 years (aHR = 2.73, 95% CI: 1.08 to 6.87) (Table 3).Comparisons of thefour groups showed an inferior patient survival rate as well renal graft survival in patients with either HBV or HCV infection and with both HBV and HCV infection, which was a statistically significant difference (p < 0.0001) (Figs 1,2). In terms of the cumulative incidence rate of overall de novo hepatic diseases and individual subtypes among these groups, kidney transplant recipients with HBV infection had higher incidence rates of HCC and hepatic failure, and higher incidence rates of liver cirrhosis with HCV infection, all of which reached statistical significance (p < 0.0001) (Fig. 3A–D).

## Discussion

In the present study, we showed an inferior outcome of kidney recipients with chronic hepatitis virus infection and subsequent risk of liver diseases in different hepatitis virus infection after transplantation. Significantly inferior patient survival was noted in transplant recipients with hepatitis virus infection as compared to those without the hepatitis virus; however, it is worth noting that survival between the two hepatitis virus infection cohorts was not statistically different. After adjusting confounders including age, sex and related cardiovascular risk factors, hepatitis virus infection (either HBV or HCV infection) was associated with an approximately 2-fold increased risk of patient mortality. The influence of virus hepatitis on kidney recipients could not be found in the previous study regarding kidney transplantation in patients with and without hepatitis virus infection in Taiwan^5^. In contrast, the detrimental effect of hepatitis virus infection on kidney transplant patients was noted in our study. This disparity may be explained by the increasing importance of the role of hepatitis virus infection after the other competing risk factors that may affect kidney transplant patient survival, including cardiovascular factors, infection, and malignancy, have been overcome or improved upon in recent years. In addition, the slow progression of subclinical liver diseases post-transplantation to the point of clinical manifestation of liver abnormality requires a longer period of time.

In the other previous studies of hepatitis virus infection and kidney transplantation, HBV infection was suggested to be significantly associated with an increased risk (adjusted relative risk, a RR = 2.214) of all-cause mortality, and HCV infection had a RR = 1.855^5^,^9^. Although there remains some controversy regarding the influence of hepatitis virus on kidney transplantation, our data demonstrated the significantly adverse impact of the hepatitis virus on kidney transplantation which was consistent with the previous findings.

We further determined the subsequent risk of post-transplant liver disease in different HBV and HCV infection patients using the Cox regression model. First, we attempted to identify the risk factors that were associated with the occurrence of post-transplant liver disease. HBV infection in kidney recipients carried a 7.08-fold increased risk, HCV infection, a 7.14-fold increased riskand both HBV and HCV infection, a 2.96-fold increased risk. We then calculated the risk of individual liver disease in kidney recipients among these groups. Our data showed that the risk of hepatitis virus in different liver diseases varied greatly in kidney transplant recipients.

Kidney recipients with HBV infection had anapproximately10-fold increased risk of developing HCC and an 7-fold increased risk of fulminant hepatic failure. In contrast, HCV cases had a dominate role of liver cirrhosis after transplantation which was with approximately 18-fold increased risk. In the data of U.S. Scientific Registry of Transplant Recipients (SRTR), virus hepatitis is found to be significantly associated with the occurrence of de novo HCC among non-liver solid organ recipients^10^. Oncogenic viral infection has been demonstrated to be critical to the pathogenesis of de novo HCC in kidney transplantation^11^. Our results support these previous findings and suggest that close monitoring of the liver condition in kidney transplant patients with HBV infection is crucial.

Whether immunosuppression would contribute to progress in HCV related liver cirrhosis in transplant recipients remains an equivocal issue^12^,^13^. A recent study exploring the influence of kidney transplantation on liver cirrhosis in 207 HCV-related ESRD patients found that kidney transplantation does not seem to accelerate liver injury; 77% of kidney recipients showed stable or improved liver biopsy results in the follow-up compared to when they were on the waiting list^12^. In the study, they suggested that the influence of immunosuppressant on liver cirrhosis seemed to be milder than we had considered previously. Furthermore, in our study, we calculated the influence of immunosuppression on post-transplant liver diseases and found that none of the immunosuppressants were associated with increased risk of post-transplant liver diseases and that this was statistically insignificant. In addition, our data showed that HCV infection was significantly associated with a strikingly high risk of liver cirrhosis post-transplantation while compared to patients without hepatitis virus. Our results supported the previous findings and highlight the critical role of HCV in contributing to post-transplant liver cirrhosis other than the effect of immunosuppression. It is much more difficult to treat HCV in transplant recipients which may result in unacceptably high rejection rates in kidney recipients. Hence, we reinforce the importance that it is imperative to eradicate HCV infection before kidney transplantation to achieve negative HCV-RNA^14^.

Lastly, we compared the risk of fulminant hepatic failure in renal recipients and showed that HBV infection had an approximately 6-fold increased risk of fulminant hepatic failure after transplantation; nevertheless, this was not found in HCV cases. Our data showed that the occurrence of fulminant hepatic failure in HBV cases seemed to be found in the early years of transplantation and declined thereafter. The rapid reactivation of HBV was associated with the use of anti-lymphocyte immunoglobulin and methylprednisolone for induction therapy and the introduction of antiviral agents for HBV, such as lamivudine in renal recipients has been achieved a great decline in the occurrence of fulminant hepatic failure in our transplant cohort^15^. To a certain degree, our data may reflect the benefits of lamivudine agent use in kidney recipients, either preemptively or as prophylactic therapy. In Taiwan, prophylaxis therapy such as anti-HBV is not routinely implemented in renal transplant recipients with chronic hepatitis virus infection and a relatively small portion of renal transplant recipients receive anti-HBV therapy. However, a significantly high risk of post-transplant liver disease remains to be observed in our renal transplant cohort. Therefore, the impact of hepatitis virus infection on post-transplant liver disease may be underestimated in the study.

The role of hepatitis virus in contributing to de novo liver disease after transplantation was clearly explored in this cohort study, but some limitations regarding the study itself should also be clarified. First, we lack data on liver biochemistry and tissue data as it could not be obtained and compared in the database utilized. In addition, the data regarding hepatitis B virus (HBV) as well as hepatitis C virus (HCV) such as virus genotype and titer cannot be obtained either. In Taiwan, ESRD patients on a waiting list are regularly monitored for their liver conditions with imaging studies (abdominal ultrasonography and computer tomography) and biochemistry examinations including GOT/GPT, albumin, alpha-fetoprotein, hepatitis B and C virus markers, and so on. Among patients undergoing maintenance dialysis, there was reluctance to perform a liver biopsy due to caution regarding uremic bleeding and platelet dysfunction. It should be highlighted that the diagnosis of every liver disease among transplant recipients was performed by specialists in the hospital and this may help to overcome the lack of certain data.

In conclusion, we report on the significant risk of de novo liver diseases in kidney transplant patients with and without hepatitis virus infection and demonstrated the adverse outcomes of kidney recipients with hepatitis virus infection. Based our findings, we would advise using caution regarding the liver condition of patients with hepatitis virus infection after transplantation.

## Additional Information

**How to cite this article**: Yu, T.-M. *et al*. Increased risk of hepatic complications in kidney transplantation with chronic virus hepatitis infection: A nationwide population-based cohort study. *Sci. Rep*. **6**, 21312; doi: 10.1038/srep21312 (2016).

## Figures and Tables

**Table 1:** Demographic and clinical information in kidney transplantation cohort classified by status of hepatitis virus infection.

		Group 1 (B–/C–)	Group 2 (B + /C–)	Group3 (B–/C + )	Group4 (B + /C + )	*P* value
Number		3485	336	262	50	
Gender						0.0036
	Female	1718 (49.3)	137 (40.8)	121 (46.2)	17 (34.0)	
	Male	1767 (50.7)	199 (59.2)	141 (53.8)	33 (66.0)	
Age at kidney transplantation						0.0031
	18 to 40 years	1076 (30.9)	112 (33.3)	67 (25.6)	6 (12.0)	
	40 to 65 years	2264 (65)	217 (64.6)	190 (72.5)	41 (82.0)	
	65 + years	145 (4.2)	7 (2.1)	5 (1.9)	3 (6.0)	
Date of kidney transplantation						0.0031
	2000 to 2005	2181 (62.6)	179 (53.3)	174 (66.4)	29 (58.0)	
	2006 to 2009	1304 (37.4)	157 (46.7)	88 (33.6)	21 (42.0)	
Comorbidity at recruitment						
	Diabetes	631 (18.1)	43 (12.8)	67 (25.6)	11 (22.0)	0.0008
	Hypertension	2419 (69.4)	243 (72.3)	180 (68.7)	32 (64.0)	0.5589
	Chronic glomerulonephritis	1148 (32.9)	133 (39.6)	79 (30.2)	14 (28.0)	0.0465
	Heart failure	249 (7.1)	12 (3.6)	21 (8.0)	4 (8.0)	0.0820
	Coronary artery disease	312 (9)	25 (7.4)	26 (9.9)	4 (8.0)	0.7330
Immunosuppresants						
		3449 (99.0)	334 (99.4)	261 (99.6)	49 (98.0)	0.5447
Cyclosporin		1645 (47.2)	155 (46.1)	176 (67.2)	32 (64.0)	<0.0001
Tacrolimus		2649 (76)	278 (82.7)	195 (74.4)	37 (74.0)	0.0366
Sirolimus		1509 (43.3)	137 (40.8)	130 (49.6)	21 (42.0)	0.1640
mycophenolatemofetil		3215 (92.3)	314 (93.5)	250 (95.4)	44 (88.0)	0.1431
Lamivudine		64 (1.8)	151 (44.9)	4 (1.5)	13 (26.0)	<0.0001
Median follow-up of mortality, years (SD)		5.6 (2.8)	4.9 (2.8)	5.6 (2.9)	5.4 (2.8)	0.0001

**Table 2:** Hazard ratios of mortality and subsequent hepatic diseasesstratified by virus status inkidney transplantation population.

	Group 1 (B–/C–)			Group 2 (B + /C–)			Group 3 (B–/C + )			Group 4 (B + /C + )		
	Event	PY	Rate	Event	PY	Rate	Event	PY	Rate	Event	PY	Rate
**Death**	335	19480	17.2	52	1631	31.9	51	1456	35.0	6	269	22.3
Crude HR			ref			1.88 (1.40–2.52)			2.04 (1.52–2.73)			1.31 (0.58–2.93)
Adjusted HR			ref			2.99 (2.13–4.18)			2.05 (1.52–2.76)			1.36 (0.61–3.07)
**Graft failure**	225	18578	12.1	24	1564	15.3	45	1323	34.0	6	244	24.6
Crude HR			ref			1.31 (0.86–2.00)			2.87 (2.08–3.95)			2.11 (0.94–4.74)
Adjusted HR			ref			1.47 (0.90–2.38)			2.49 (1.79–3.44)			2.32 (1.02–5.28)
**Overall hepatic diseases**	68	19330	3.52	46	1552	29.6	35	1393	25.1	4	256	15.7
Crude HR			ref			8.35 (5.74–12.2)			7.16 (4.76–10.8)			4.41 (1.61–12.1)
Adjusted HR			ref			7.08 (4.43–11.3)			7.14 (4.70–10.9)			2.96 (1.06–8.24)
**Hepatocellular carcinoma (ICD 155)**	23	19330	1.19	17	1552	11.0	7	1393	5.03	2	256	7.83
Crude HR			ref			9.22 (4.92–17.3)			4.25 (1.82–9.91)			6.53 (1.54–27.7)
Adjusted HR			ref			9.84 (4.61–21.0)			4.40 (1.85–10.5)			4.63 (1.06–20.2)
**Liver cirrhosis (ICD 571.5)**	21	19330	1.09	15	1552	9.67	24	1393	17.2	0	256	0
Crude HR			ref			8.92 (4.59–17.3)			15.9 (8.87–28.6)			—
Adjusted HR			ref			5.86 (2.42–14.2)			18.0 (9.78–33.2)			—
**Hepatic failure (ICD 570)**	24	19330	1.24	14	1552	9.02	4	1393	2.87	2	256	7.83
Crude HR			ref			7.01 (3.62–13.6)			2.30 (0.80–6.62)			6.18 (1.46–26.1)
Adjusted HR			ref			5.63 (2.47–12.8)			2.06 (0.71–6.00)			4.6 (1.05–20.1)

Rate was calculated per 1000 person-years. Overall hepatic diseases including hepatocellular carcinoma (ICD-9 code 155.0), hepatic failure (ICD 570), andliver cirrhosis (ICD 571.5). Adjusted HRs were adjusted for age, sex, diabetes mellitus, hypertension, heart failure, coronary artery disease, chronic glomerulonephritis, Sirolimus, mycophenolate, Cyclosporin, Tacrolimus and Lamivudine.

**Table 3:** Predictors of mortality and subsequent hepatic disease in kidney transplantation population.

	Death		Overall liver diseases	
	Crude HR	Adjusted HR	Crude HR	Adjusted HR
Hepatitis virus status				
Group 1 (B–/C–)	ref	ref	ref	ref
Group 2 (B + /C–)	1.88 (1.40–2.52)	2.90 (2.07–4.05)	8.35 (5.74–12.2)	6.76 (4.24–10.8)
Group 3 (B–/C + )	2.04 (1.52–2.73)	1.98 (1.47–2.67)	7.16 (4.76–10.8)	6.57 (4.31–10.0)
Group 4 (B + /C + )	1.31 (0.58–2.93)	1.32 (0.58–2.97)	4.41 (1.61–12.1)	2.82 (1.01–7.88)
Gender				
Female	ref	ref	ref	ref
Male	1.30 (1.08–1.57)	1.23 (1.01–1.48)	1.90 (1.36–2.66)	1.83 (1.30–2.58)
Age at kidney transplantation				
18 to 40 years	ref	ref	ref	ref
40 to 65 years	2.64 (2.04–3.42)	2.48 (1.91–3.24)	2.60 (1.69–3.99)	2.69 (1.73–4.18)
65 + years	6.14 (4.15–9.10)	5.63 (3.74–8.48)	2.43 (1.00–5.93)	2.73 (1.08–6.87)
Comorbidity				
Diabetes				
No	ref	ref	ref	ref
Yes	1.82 (1.47–2.26)	1.34 (1.07–1.69)	0.99 (0.64–1.52)	0.84 (0.53–1.34)
Hypertension				
No	ref	ref	ref	ref
Yes	1.14 (0.93–1.40)	1.01 (0.82–1.24)	0.69 (0.50–0.95)	0.60 (0.43–0.84)
Chronic glomerulonephritis				
No	ref	ref	ref	ref
Yes	0.87 (0.71–1.07)	0.84 (0.68–1.04)	0.76 (0.53–1.09)	0.86 (0.59–1.27)
Congestive Heart failure				
No	ref	ref	ref	ref
Yes	1.81 (1.33–2.46)	1.76 (1.28–2.41)	1.55 (0.90–2.69)	1.72 (0.97–3.04)
Coronary artery disease				
No	ref	ref	ref	ref
Yes	1.99 (1.52–2.61)	1.39 (1.04–1.85)	1.6 (0.98–2.62)	1.42 (0.84–2.41)
Cyclosporin use				
No	ref	ref	ref	ref
Yes	1.18 (0.97–1.42)	1.02 (0.81–1.27)	1.18 (0.85–1.63)	1.27 (0.88–1.83)
Tacrolimus use				
No	ref	ref	ref	ref
Yes	0.8 (0.65–0.98)	0.81 (0.64–1.03)	1.41 (0.94–2.11)	1.46 (0.93–2.31)
Sirolimus				
No	ref	ref	ref	ref
Yes	1.09 (0.91–1.32)	1.20 (0.99–1.45)	0.89 (0.65–1.23)	0.82 (0.59–1.13)
mycophenolate				
No	ref	ref	ref	ref
Yes	0.72 (0.53–0.98)	0.73 (0.53–1.01)	0.55 (0.34–0.89)	0.47 (0.29–0.78)
Lamivudine				
No	ref	ref	ref	ref
Yes	0.84 (0.54–1.30)	0.48 (0.29–0.79)	4.28 (2.87–6.38)	1.59 (0.95–2.65)

Adjusted HRs were adjusted for age, sex, diabetes mellitus, hypertension, heart failure, coronary artery disease, chronic glomerulonephritis, Sirolimus, mycophenolate, Cyclosporin,Tacrolimus and Lamivudine.
